# Perceptual Biases in Relation to Paranormal and Conspiracy Beliefs

**DOI:** 10.1371/journal.pone.0130422

**Published:** 2015-06-26

**Authors:** Michiel van Elk

**Affiliations:** 1 Department of Psychology, University of Amsterdam, Amsterdam, the Netherlands; 2 Amsterdam Brain and Cognition Centre, University of Amsterdam, Amsterdam, the Netherlands; University of Akron, UNITED STATES

## Abstract

Previous studies have shown that one’s prior beliefs have a strong effect on perceptual decision-making and attentional processing. The present study extends these findings by investigating how individual differences in paranormal and conspiracy beliefs are related to perceptual and attentional biases. Two field studies were conducted in which visitors of a paranormal conducted a perceptual decision making task (i.e. the face / house categorization task; Experiment 1) or a visual attention task (i.e. the global / local processing task; Experiment 2). In the first experiment it was found that skeptics compared to believers more often incorrectly categorized ambiguous face stimuli as representing a house, indicating that disbelief rather than belief in the paranormal is driving the bias observed for the categorization of ambiguous stimuli. In the second experiment, it was found that skeptics showed a classical ‘global-to-local’ interference effect, whereas believers in conspiracy theories were characterized by a stronger ‘local-to-global interference effect’. The present study shows that individual differences in paranormal and conspiracy beliefs are associated with perceptual and attentional biases, thereby extending the growing body of work in this field indicating effects of cultural learning on basic perceptual processes.

## Introduction

Several studies have shown that culture and religion can have a strong impact on basic perceptual processes [1,2,3,4,5]. It has been found for instance that people in Asian cultures are characterized by a more holistic processing style (i.e. paying more attention to global compared to local visual features) compared to people from North America or Europe [2]. This finding has been related to the effect of culturally learned experiences, as Eastern cultures place more emphasis on social interdependence and collective identity [4]. It has also been reported that being raised in a Protestant compared to a Catholic environment is associated with a stronger local compared to a global visual processing style, and this has also been associated with the effects of learning and socialization (e.g. the Protestant ethic of individual responsibility and ‘sphere sovereignty’; [1,5]). Other studies have shown that one’s prior beliefs can have a strong effect on perceptual detection tasks (i.e. making a decision about the presence or absence of a specific sensory stimulus). Paranormal beliefs are strongly related to perceptual biases for detecting illusory patterns for instance [6,7,8,9,10,11]. These biases have also been explained as reflecting culture-specific learning experiences [10,11]. The ability to detect and interpret patterns in noisy data is central to many superstitious and paranormal beliefs (e.g. palm reading, tasseography etc.) and believers likely have profound experience with detecting illusory patterns, thus affecting their performance on perceptual detection tasks.

As these examples illustrate, perceptual and attentional processing are strongly influenced by prior expectations and learning experiences. The present study aimed to replicate and extend these findings by focusing on the perceptual and attentional biases that are associated with belief in the paranormal and belief in conspiracy theories. More specifically, the present study advances our understanding of the relation between prior beliefs and perceptual processing in two important ways.

First, previous studies on the relation between paranormal beliefs and perceptual processing have typically used perceptual detection tasks in which participants were required to detect the presence or absence of a specific stimulus (e.g. a face or a word; cf. [6,7,8,9,10,11]). In these studies by using a signal detection analysis it was found that paranormal believers showed a response bias toward detecting illusory patterns in random noise. However, based on these findings, it remains unclear whether paranormal believers show a domain-general bias (i.e. a ‘yes’-saying tendency) or rather a domain-specific bias toward detecting specific stimulus categories. This issue is of central importance to theories about the cognitive basis of paranormal and religious beliefs, as it is often argued that these beliefs are primarily related to biases toward detecting illusory agency (i.e. inferring the presence of non-existent agents; [10,11,12,13,14]). That is, evolutionary accounts of religious and supernatural beliefs have proposed that a tendency to over-detect the presence of other agents may be at the basis of belief in supernatural agents [12,13,15,16]. The false positives generated by the so-called ‘hyperactive agency detection device’ may reinforce the belief that invisible and omnipresent agents actually exist. Following this line of reasoning, one may expect that believers compared to skeptics show a specific bias toward detecting the presence of other agents. Thus, in the first experiment presented in this paper a direct comparison was made between the detection of *human agents* versus *non-natural objects* (i.e. by using a face / house categorization task [17]) and the relation with paranormal beliefs was investigated. Visitors of a psychic fair were required to conduct a visual categorization task, by classifying pictures with different levels of visual noise as representing a face or a house. By comparing the number of incorrectly classified face- and house-pictures between believers and skeptics, it could be investigated whether the previously reported association between paranormal beliefs and illusory agency detection [10,11] reflects a domain-specific mechanism (i.e. related to the illusory detection of agency) or a domain-general phenomenon (i.e. a more liberal response bias for believers compared to skeptics).

Second, in addition to focusing on paranormal beliefs the present study also investigated the relation between attentional biases and belief in conspiracy theories (e.g. about the 9–11 attack on the WTC, the death of John F. Kennedy or Princess Diana, or UFOs, cf. [18]). Previous studies have shown that belief in conspiracy theories is related to existential threat and uncertainty [19,20,21] and other studies have shown that both motivational and social factors can have a strong effect on the endorsement of belief in conspiracy theories [22,23]. In addition, several studies have shown a strong link between paranormal beliefs and belief in conspiracy theories [18,24,25]. Interestingly, a recent study suggested that similar cognitive biases that play a role in paranormal beliefs, such as an anthropomorphistic tendency to over-attribute agency is associated with belief in conspiracy beliefs as well [26]. Given the strong link between paranormal and conspiracy beliefs, in the present study it was investigated to what extent attentional biases to selectively attend to either global or local visual information [1,5], are selectively associated with a specific belief type (i.e. paranormal or conspiracy beliefs) or whether perceptual biases generalize across beliefs.

Based on the existing literature two opposing predictions could be made regarding the relation between paranormal and conspiracy beliefs and global / local visual processing. On the one hand, belief in conspiracy theories and paranormal beliefs as well have been related to a monological belief system (i.e. a unitary closed-off world view) in which events are selectively being viewed as reflecting the intended outcome of evil powers or forces [27]. On this account one could expect that paranormal and conspiracy beliefs are related to a local compared to a global processing bias, as contextual information is selectively discarded (i.e. a ‘tunnel vision’). Alternatively, it could be argued that paranormal and conspiracy beliefs are related to a stronger global compared to a local processing bias, as believers typically tend to see the ‘bigger picture’ and tend to associate unrelated events. It has been found for instance, that believers in conspiracy theories often hold apparently contradictory views (e.g. that Princess Diana was killed by the MI6) that are in fact both compatible with a broader conspiracist worldview [28]. In addition, it has been reported that analytic compared to intuitive thinking is associated with a reduced belief in conspiracy theories (e.g. the belief that governments intentionally withhold important information from the general public; [29,30]). Thus, given the exploratory nature of this study it was investigated whether conspiracy and paranormal beliefs were related to either a global or a local processing bias. To this end, the second study reported in this paper used a Navon task in which participants were required to selectively report either the global or the local shape of geometrical figures [31]. The geometrical figures were composed of smaller shapes, which could be congruent or incongruent with respect to the global shape. Typically, participants respond faster when attending to global compared to local information, which is known as the global-precedence effect [32]. In addition, information from the irrelevant dimension may interfere with visual attentional processing, resulting in a global-to-local interference effect (whereby incongruent global information interferes with reporting the local shapes), which is typically stronger in size than the local-to-global interference effect (whereby incongruent local information interferes with reporting the global shapes).

In sum, in two experiments the perceptual and attentional biases associated with paranormal and conspiracy beliefs were investigated, by means of a perceptual decision making task (i.e. the face / house categorization task) and a visual attention task (i.e. the global / local task). Both experiments were conducted as a field study at a paranormal fair. Typically, visitors of a paranormal fair differ strongly in their paranormal and conspiracy beliefs, ranging from strong paranormal believers (i.e. often visiting a spiritual medium) to skeptics (i.e. people who are merely interest in seeing what is going on). By including well-established questionnaires to assess individual differences in prior beliefs, it was possible to test people with different backgrounds on two classical perceptual processing tasks under similar experimental circumstances.

## Materials and Methods

### Experiment 1: Materials and Methods

#### Participants

A total of 55 (17 men, mean age = 43.3 years) participants with normal or corrected-to-normal vision were included in the first experiment. All participants were recruited at a paranormal fair (i.e. Paraview, Spijkenisse, the Netherlands; www.paraview.nl; Paranormaal Alternatief, Rijswijk, the Netherlands; http://www.paranormaalalternatief.nl/). Visitors of the fair were asked to participate in a study. Due to the field setting of these experiments, participants completed the task in a rather noisy environment compared to lab-based studies. We note that this situation was the same for all participants that participated in these studies. All participants gave written informed consent and were offered a financial remuneration for participation (3.50 Euros). The study protocol was approved by the local ethics committee at the University of Amsterdam (i.e. the Ethics Review Board of the Faculty of Social and Behavioural Sciences; Project code: 2013-SP-2725). The experiment was conducted in accordance with the guidelines of the Declaration of Helsinki.

#### Stimuli

**Fig 1:**
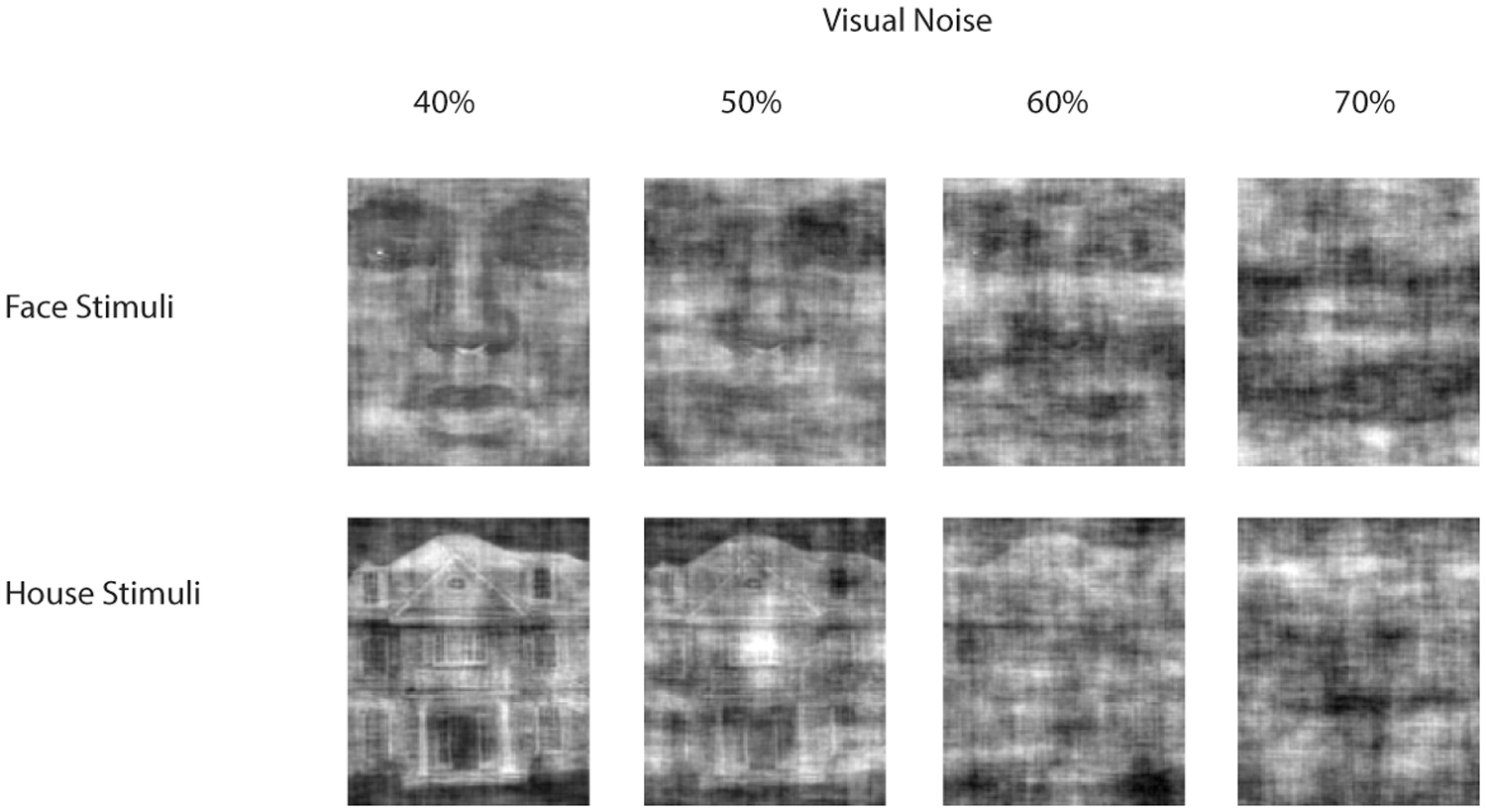
Example stimuli used in the experiment representing faces (upper row) or houses (lower row) with increased levels of visual noise from left to right.

As stimuli a selection of pictures was used from the original stimulus set that has been developed to study the neural correlates of perceptual decision making [17]. The set consisted of 38 black and white pictures of faces and houses (131 x 156 pixels), to which different levels of random visual noise had been added (see Fig 1 for example stimuli). For the present study stimuli representing 40%, 50%, 60% and 70% of visual noise were selected. In a previous study it has been found that the average threshold for 82% correct was at 45% visual noise [17]. Accordingly, by using the range from 40–70% it was ensured that for the lowest level of visual noise most participants would be able to correctly classify most stimuli, whereas with an increased level of visual noise perceptual categorization would become more difficult.

#### Experimental setup and procedure

The experiment was conducted at two paranormal fairs, which took place in big sports halls in the suburbs of Dutch cities. Four laptop computers (Dell Latitude E6140) were placed on a long table covered by a black cloth. Participants were seated behind the table and after signing the consent form the experimental task was explained. Participants were instructed that they were going to see pictures representing either a face or a house, under different levels of visual noise. They were required to indicate whether they believed the picture represented a face or a house by pressing one of two buttons on a computer keyboard (i.e. left and right arrow key). It was emphasized that if participants were uncertain about what the picture represented, that they should trust their first intuition and not think too long. The laptop was placed in front of the participant at a viewing distance of approximately 60 cm.

At the beginning of the experiment, participants conducted 10 practice trials to familiarize with the task. Each picture was presented for 3500 ms or until a response had been made. The inter-stimulus interval was 1000 ms. In total the experiment consisted of 240 trials according to the following design: 4 Noise Levels (40, 50, 60 and 70%), 2 Stimulus Categories (Faces vs. Houses) and 30 repetitions per category. The experiment was programmed using Presentation software (Neurobehavioral systems, Albany, CA, USA). At the end of the experiment, participants completed the revised paranormal belief scale (RPBS; [33])and indicated their highest level of education. The rationale for measuring paranormal beliefs following the experimental task was mainly practical: following completion of the computer task, participants could fill in the questionnaire forms at their own pace, while the laptop could be used for the next participant. In total, the experiment took about 20–30 minutes.

#### Data analysis

The accuracy data (i.e. proportion of correctly classified stimuli) were subjected to a repeated measures ANOVA with Stimulus (Face vs. House stimuli) and Noise (4 levels: 40, 50, 60 and 70%) as within-subjects factors and the score on the RPBS as a covariate. Some participants did not complete all questions of the RPBS and these responses were coded as missing values. Still for all participants an average score for the RPBS was calculated based on their total number of completed responses. To directly assess the relation between the performance on the face / house categorization task and paranormal beliefs, a regression analysis was conducted in which the effect of paranormal beliefs on accuracy was investigated, after controlling for demographic variables (i.e. gender, age and education).

### Experiment 2: Materials and Methods

#### Participants

In the second study a total of 60 (22 men, mean age = 42.4 years) participants with normal or corrected-to-normal vision were included. Three participants were excluded from analysis because they did not complete the RPBS and / or the conspiracy belief questionnaires (CBQ). All participants were recruited at a paranormal fair (i.e. Paranormaal Alternatief, Rijswijk, the Netherlands; http://www.paranormaalalternatief.nl/). Visitors of the fair were asked to participate in a study. All participants gave written informed consent and were offered a financial remuneration for participation (3.50 Euros). The study protocol was approved by the local ethics committee at the University of Amsterdam (i.e. the Ethics Review Board of the Faculty of Social and Behavioural Sciences; Project code: 2013-SP-2725). The experiment was conducted in accordance with the guidelines of the Declaration of Helsinki.

#### Stimuli

**Fig 2:**
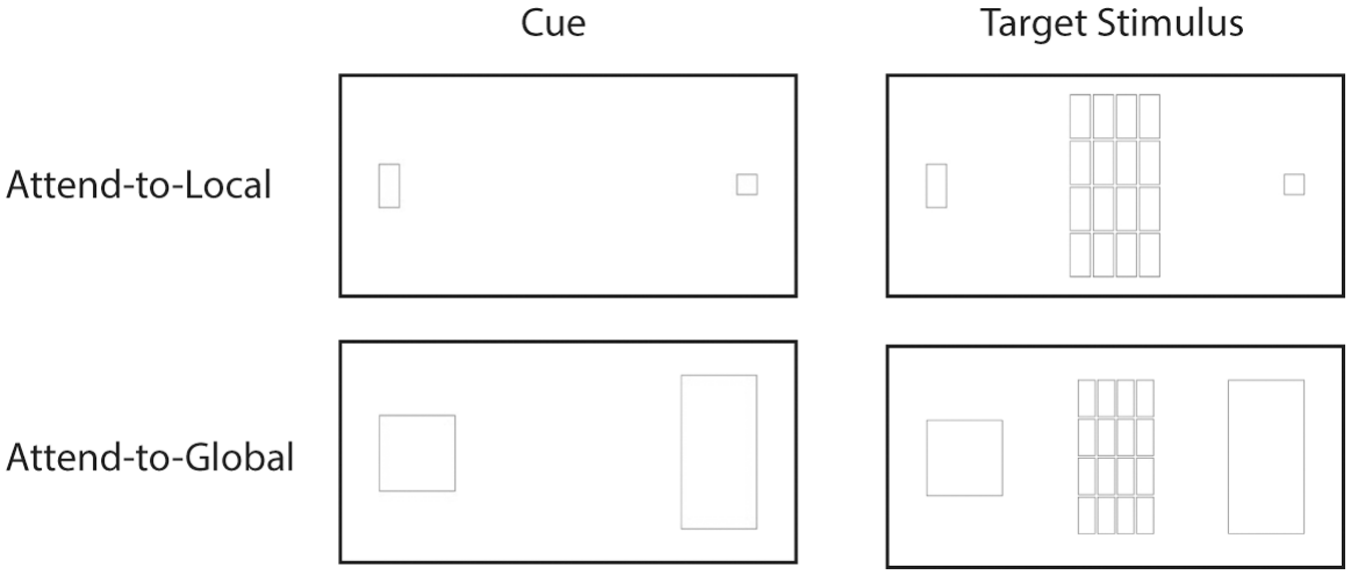
Stimuli used for the global—local processing task. Participants were first presented with a cue, indicating whether they should attend to global or to local features and the response mapping was displayed. Next, the target stimulus appeared on the screen, consisting of triangles or squares. Depending on the block and the instruction participants were required to report either the global or the local shape of the target stimulus.

As stimuli we used pictures representing geometrical figures (see Fig 2; for similar stimuli and procedure, see: [1,5]). Target stimuli consisted of large squares and rectangles that were composed of smaller squares or rectangles. A large rectangle was composed of either 4 x 8 small squares or 4 x 4 small rectangles. A large square was composed of either 4 x 4 small squares or 2 x 4 small rectangles. Thus, in total 4 different target stimuli were constructed in which the global and local features could be congruent or incongruent. All stimuli were presented at a resolution of 464 x 190 pixels.

In addition to the target stimuli, a cue stimulus was used in which either a small square and rectangle or a large square and rectangle were presented on both sides of the screen. The cue stimulus always preceded the presentation of the centrally presented target stimulus and served as a reminder of the task (‘attend to global’ vs. ‘attend to local’) and of the mapping of the response buttons (see below).

#### Experimental setup and procedure

Participants were instructed that they were going to see pictures representing geometrical figures that were composed of smaller figures. An example stimulus was shown to the participants and it was pointed out that each figure could be characterized by its global shape or its local shape. It was explained that in different blocks they were required to report either the global or the local shape of the figure by pressing two buttons. One group of participants responded with the left button to squares and with the right button to rectangles and for the other group of participants the mapping of response buttons was reversed.

Participants were instructed that in different blocks they were required to report either the global or the local shape of the figure. Each block was preceded by an explicit instruction on the screen reminding participants of whether the next block was an ‘attend-to-global’ or an ‘attend-to-local’ block. Participants were told that preceding the presentation of each fig a short cue would be presented for 500 ms. The size of the cue also indicated whether they were required to attend to the global or the local features of the central figure and the shape of the cue served as a reminder of the button that was to be used for categorizing squares and rectangles. The target stimulus remained on the screen for 3500 ms or until a response had been made and the inter-stimulus interval was 1000 ms. Before the start of each block participants conducted 16 practice trials to familiarize with the task. After it was established that the task was clearly understood, participants conducted 48 experimental trials. In total the experiment consisted of 4 experimental blocks and block-order (global vs. local) was counterbalanced across participants.

The experiment was programmed using Presentation software (Neurobehavioral systems, Albany, CA, USA). At the end of the experiment, participants completed the RPBS, the Conspiracy Belief Questionnaire (CBQ; [18]) and indicated their highest level of education. In total, the experiment took about 20–30 minutes.

#### Data analysis

For the analysis of the reaction time data, first all practice trials, all trials with incorrect responses or trials in which the reaction time exceeded the subject’s mean by more than two standard deviations were removed. Data was categorized according to whether participants attended to the global or the local stimulus shape and according to whether the irrelevant shape was congruent or incongruent with respect to the relevant shape. The averaged reaction times were analyzed using a repeated measures ANOVA with the within-subjects factors Attention (Global vs. Local) and Congruency (Congruent vs. Incongruent) and the average scores on the RPBS and the CBQ were included as a covariate. Some participants did not complete all questions of the RPBS and the CBQ and these responses were coded as missing values. For these participants the averaged score was calculated using the total number of completed responses. To directly assess the relation between attentional processing and conspiracy beliefs, a regression analysis was conducted in which the effect of paranormal and conspiracy beliefs on attentional biases was investigated, after controlling for demographic variables (i.e. gender, age and education). The data from both experiments (behavioral and questionnaire data) is included as (S1 File).

## Results

### Results Experiment 1: Face / House Categorization Task

#### Paranormal Beliefs

The overall average score on the RPBS was 3.8 (SD = 1.2) and Cronbach’s alpha was .92. A Shapiro-Wilk test indicated that the scores on the RPBS were normally distributed. After conducting a median split, a group of skeptics (n = 26; 11 males; mean age = 42.5 years; mean RPBS score = 2.8, SD = .9) and a group of believers (n = 29; 15 males; mean age = 42.3 years; mean RPBS score = 4.7, SE = .6) were defined. The groups differed in level of education, *t*(53) = 2.6, *p* = .01 (mean level of education skeptics = 3.9, SE = .3, mean level of education believers = 3.0, SE = .2).

#### Accuracy

**Fig 3:**
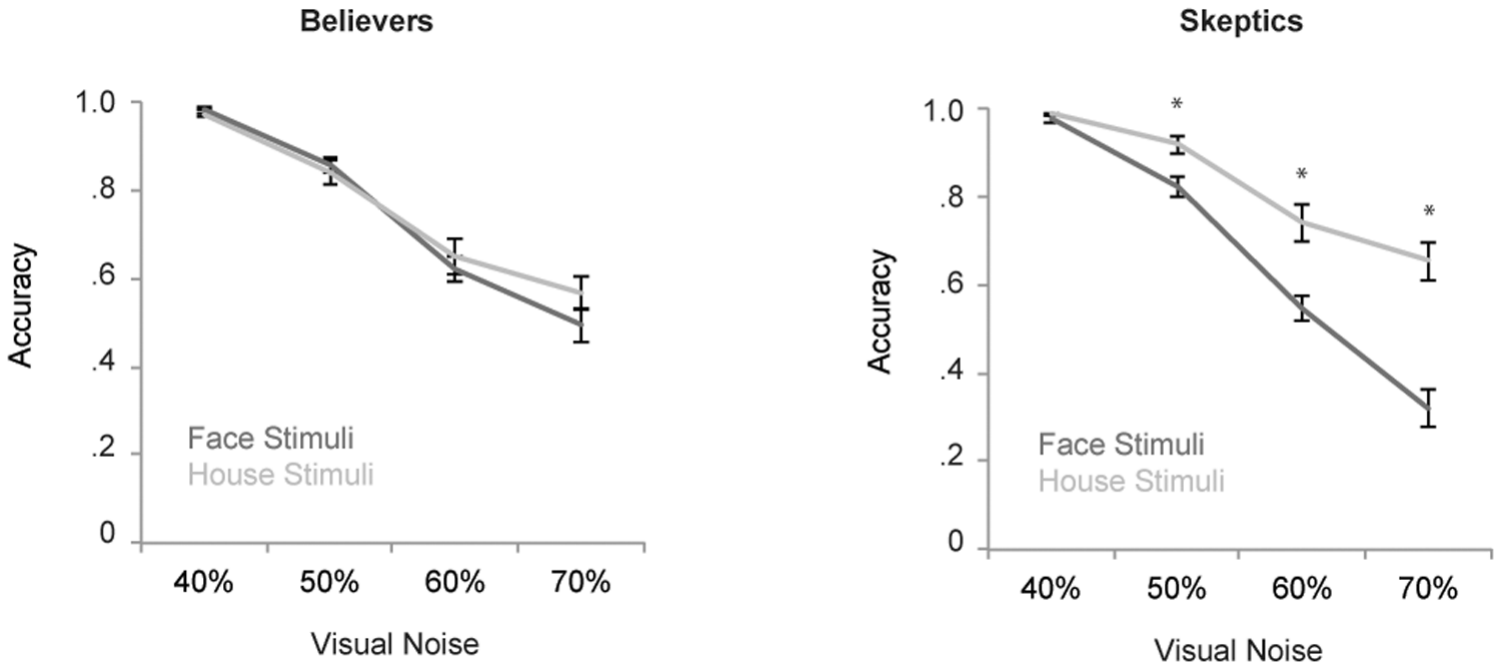
Accuracy for the face / house categorization task. The left graph represents the accuracy for believers and the right graph for skeptics. The x-axis represents the different levels of visual noise (40%, 50%, 60% and 70%). Dark lines represent responses to face stimuli and light lines represent responses to house stimuli. Error bars represent standard errors.

The accuracy in categorization responses as a function of stimulus type is represented in Fig 3. A main effect of Stimulus was found, *F*(1, 53) = 16.0, *p* < .001, *η* ^*2*^ = .23, indicating a higher accuracy for the categorization of house stimuli (mean accuracy = .79) compared to face stimuli (mean accuracy = .70). An interaction between Stimulus and the RPBS score was found, *F*(1, 53) = 10.8, *p* < .001, *η* ^*2*^ = .17, reflecting that the effect of stimulus on accuracy was modulated by the score on the RPBS. As expected, a main effect of Noise was found, *F*(3, 159) = 81.0, *p* < .001, *η* ^*2*^ = .60, indicating that with increased levels of visual noise the accuracy decreased (see Fig 3). Noise also interacted with the score on the RPBS, *F*(3,159) = 3.8, *p* < .05, *η* ^*2*^ = .07. These main effects were qualified by a significant interaction between Stimulus and Noise, *F*(3, 159) = 11.8, *p* < .001, *η* ^*2*^ = .18 and an interaction between Stimulus, Noise and the RPBS score, *F*(3, 159) = 6.9, *p* < .001, *η* ^*2*^ = .12.

As can be seen in Fig 3, this three-way interaction reflects that whereas skeptics showed a differential response to face compared to house stimuli with increased levels of visual noise, for believers the accuracy for face and house stimuli was not modulated as a function of visual noise. To explore this three-way interaction, two separate ANOVAs were conducted for the group of skeptics and the group of believers (for recommendations on the interpretation of three-way interactions in mixed design ANOVAs, see: [34]). For skeptics we observed a significant main effect of Stimulus, *F*(1, 25) = 12.8, *p* < .001, *η* ^*2*^ = .34, a main effect of Noise, *F*(3, 75) = 379.6, *p* < .001, *η* ^*2*^ = .94 and a significant interaction between Stimulus and Noise, *F*(3, 75) = 11.7, *p* < .001, *η* ^*2*^ = .32. Post-hoc t-tests indicated that the accuracy was lower for face compared to house stimuli with 50% noise, *t*(25) = 2.9, *p* < .01, with 60% noise, *t*(25) = 2.7, *p* < .05 and with 70% noise, *t*(25) = 4.1, *p* < .001 (see Fig 3). In contrast, for believers only a main effect of Noise was observed, *F*(3, 84) = 270.6, *p* < .001, *η* ^*2*^ = .91 and the interaction between Stimulus and Noise was not significant (*F* = 1.0, n.s.). When the difference between responses to face and house stimuli between both groups were directly compared, it was found that only stimuli with 70% of visual noise would survive Bonferroni correction for multiple comparisons, *t*(53) = 2.6, *p* = .012.

#### Regression Analysis

In a linear regression analysis the effects of demographic variables (age, gender and education) and paranormal beliefs on the accuracy was determined. We focused specifically on face stimuli with 70% of visual noise, as this was the stimulus category for which both groups differed in their responses to face and house stimuli. As can be seen in Table 1, age and education were a small though significant predictor of the accuracy: with increased age the accuracy increased and higher levels of education were associated with a decreased accuracy. As expected, the RPBS was a significant predictor of the accuracy, while controlling for the demographic variables.

**Table 1:** Hierarchical multiple regression analyses predicting the accuracy for face stimuli with 70% visual noise.

		B	S.E.	b	t	p
Step 1						
	Intercept	0.296	0.187		1.579	0.121
	Age	0.005	0.002	0.281	2.218	0.031
	Gender	0.068	0.063	0.14	1.081	0.285
	Education	-0.058	0.023	-0.331	-2.562	0.014
Step 2						
	Intercept	0.111	0.19		0.584	0.562
	Age	0.004	0.002	0.225	1.847	0.071
	Gender	0.021	0.062	0.044	0.342	0.734
	Education	-0.04	0.022	-0.227	-1.774	0.082
	RPBS	0.002	0.001	0.353	0.264	0.011

Note: ΔR^2^ = .23 for Step 1; ΔR^2^ = .33 for Step 2.

### Results Experiment 2: Global / local processing task

#### Paranormal and Conspiracy Beliefs

The overall average score on the RPBS was 4.0 (SD = 1.0) and Cronbach’s alpha was .81. For the CBQ the average score was 4.5 (SD = 2.2) and Cronbach’s alpha was .96. Shapiro-Wilk tests indicated that the scores on both scales were normally distributed. The RPBS and the CBQ were moderately correlated, *r* = .444, *p* = .001. After conducting a median split on the CBQ score, a group of conspiracy skeptics (n = 28; 9 males; mean age = 45.5 years; mean RPBS score = 3.6, SD = 1.2; mean CBQ score = 2.9, SD = 1.0) and a group of believers in conspiracy theories (n = 29; 11 males; mean age = 46.0 years; mean RPBS score = 4.3, SD = .7; mean CBQ score = 6.2, SD = 1.7) were defined. The groups differed in their level of education, t(55) = 2.1, p = .044 (mean level of education skeptics = 4.3, SE = .2, mean level of education believers = 3.7, SE = .2).

#### Reaction Times

**Fig 4:**
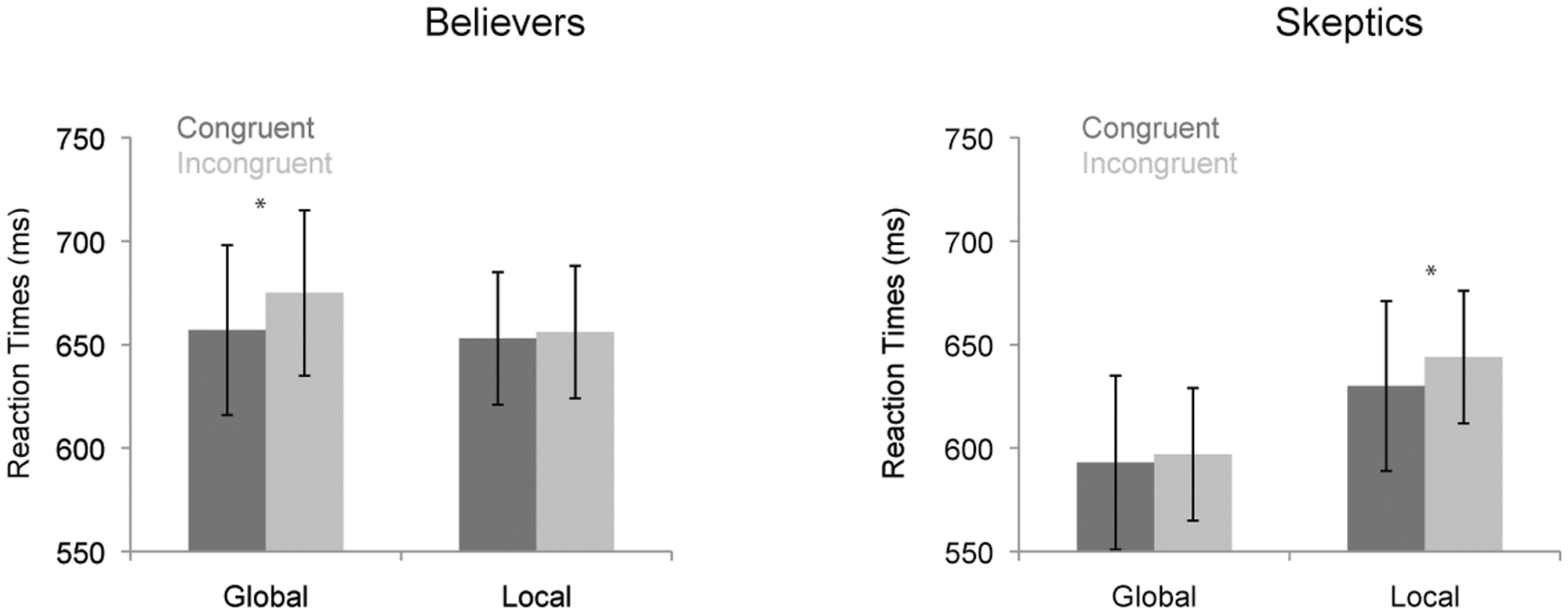
Reaction times for the global / local processing task. The left graph represents responses for believers in conspiracy theories and the right graph for skeptics. Left bars represent responses during ‘attend-to-global’ blocks and right bars for ‘attend-to-local’ blocks. Dark bars represent congruent and light bars incongruent trials. Error bars represent standard errors.

Reaction times are presented in Fig 4. Analysis of the reaction times indicated a main effect of Congruency, *F*(1, 56) = 12.4, *p* < .001, *η* ^*2*^ = .18, reflecting that participants responded faster on congruent (mean RT = 631 ms, SE = 29) than on incongruent trials (mean RT = 645 ms, SE = 22). No other effects were found significant. Including the RPBS as a covariate in the analysis did not reveal significant interactions between the experimental factors and paranormal beliefs (*F* < 1). Including the CBQ as a covariate in the analysis revealed an interaction between Attention, Congruency and the CBQ, *F*(1, 55) = 9.8, *p* < .005, *η* ^*2*^ = .15.

To explore this three-way interaction two separate ANOVAs were conducted for the group of skeptics and believers. For skeptics a marginally significant interaction was found between Attention and Congruency, *F*(1, 27) = 4.0, *p* .055, *η* ^*2*^ = .13, indicating that the congruency effect (i.e. RT difference between incongruent and congruent trials) was stronger on local trials (ΔRT = 14 ms; t(27) = 3.3, p < .005) compared to global trials (ΔRT = 3 ms; n.s.). For believers, the interaction between Attention and Congruency was not significant, F(1, 28) = 3.0, p = .095, *η* ^*2*^ = .10, but the directionality was opposite that observed for skeptics: the congruency effect was stronger for global trials (ΔRT = 17 ms; t(28) = 3.1, p < .005) compared to local trials (ΔRT = 3 ms; n.s.). Thus, skeptics showed a classical global-to-local interference effect, whereas believers showed a stronger local-to-global interference effect.

#### Regression analysis

In two regression analyses we directly investigated the effect of belief on conspiracy beliefs on the global-to-local interference effect and the local-to-global interference effect, while controlling for demographic variables (gender, age and education) and for paranormal beliefs. As can be seen in Table 2, the CBQ score was negatively associated with the global-to-local interference effect (i.e. the RT difference between incongruent and congruent trials during attend-to-local blocks): whereas people scoring low on conspiracy beliefs showed a moderate global-to-local interference effect, this effect was reduced for people scoring high on conspiracy beliefs. Table 3 represents the analysis of the local-to-global interference effect (i.e. the RT difference between incongruent and congruent trials during attend-to-global blocks): the CBQ score was positively associated with the local-to-global interference effect, indicating that believers were characterized by a stronger interference from irrelevant local information.

**Table 2:** Hierarchical multiple regression analyses predicting the global-to-local interference effect (ΔRT Incongruent Local—Congruent Local trials).

		B	S.E.	b	t	p
Step 1						
	Intercept	-6.23	26.36		-2.36	0.814
	Age	-0.1	0.27	-0.01	-0.04	0.97
	Gender	-1.1	8.47	-0.02	-0.13	0.9
	Education	4.3	3.61	0.16	1.18	0.242
Step 2						
	Intercept	-9.54	29.21		-0.33	0.75
	Age	-0.15	0.28	-0.08	-0.56	0.581
	Gender	-6.39	8.97	-0.11	-0.71	0.48
	Education	5.91	3.75	0.23	1.58	0.12
	RPBS	7.83	5.13	0.28	1.53	0.13
	CBQ	-4.23	2.07	-0.31	-2.04	0.046

Note: ΔR^2^ = .17 for Step 1; ΔR^2^ = .33 for Step 2.

**Table 3:** Hierarchical multiple regression analyses predicting the local-to-global interference effect (ΔRT Incongruent Global—Congruent Global trials).

		B	S.E.	b	t	p
Step 1						
	Intercept	10.11	23.5		0.43	0.669
	Age	-0.29	0.24	-0.16	-1.21	0.23
	Gender	0.08	7.55	0	0.01	0.99
	Education	3.21	3.22	0.14	1	0.323
Step 2						
	Intercept	-4.3	25.36		-0.17	0.866
	Age	-0.24	0.24	-0.14	-0.99	0.325
	Gender	2.04	7.8	0.04	0.26	0.76
	Education	2.93	3.25	0.13	0.9	0.372
	RPBS	-2.85	4.56	-0.11	-0.64	0.526
	CBQ	4.73	1.8	0.39	2.63	0.011

Note: ΔR^2^ = .22 for Step 1; ΔR^2^ = .41 for Step 2.

## Discussion

In two field experiments conducted at a paranormal fair it was investigated how paranormal and conspiracy beliefs are related to perceptual and attentional biases. The aim of these studies was to investigate (1) whether paranormal beliefs are specifically associated with a bias toward detecting agents compared to non-agents and (2) whether conspiracy beliefs are associated with an attentional bias to either local or global information.

In the first experiment it was found that paranormal beliefs were indeed related to a response bias for ambiguous stimuli. However, the effect was in an unexpected direction: skeptics compared to believers more often incorrectly classified an ambiguous face stimulus as representing a house. In contrast, believers performed at chance level when categorizing ambiguous face and house stimuli. This finding indicates that disbelief rather than belief in the paranormal is driving the bias observed for the categorization of ambiguous stimuli. Whereas previous studies have reported that paranormal believers show a bias toward detecting illusory agency [10,11], these studies did not contrast agent vs. non-agent stimuli. The present study indicates that when paranormal believers are directly asked to categorize a stimulus as representing an agent or a non-agent, no bias toward agency detection is observed. The agency detection bias observed in previous studies may be related to a general liberal response criterion for believers compared to skeptics (i.e. a ‘yes’-saying tendency; cf. [35]).

The absence of an agency-detection bias for paranormal believers when using a categorization rather than a detection task poses an important challenge for theories relating supernatural beliefs to perceptual biases toward detecting agency [12,13,15,16]. According to evolutionary accounts of religion, a hyperactive agency detection device (HADD) may have conferred an adaptive advantage by over-estimating the presence of other agents in the environment. It has been argued that the false positives generated by the HADD could be at the basis of belief in supernatural agents (e.g. God, demon, angels etc.). So far this evolutionary claim about the origin of supernatural beliefs lacks clear empirical support (see also: [14]) and the present study adds to this debate. Still, the finding that skeptics were more conservative in classifying ambiguous stimuli as representing faces indicates that prior beliefs influence perceptual decision making (cf. [1,5]). In the absence of clear perceptual features characteristic of faces (i.e. eyes and mouth), non-believers were more reluctant to categorize a stimulus as representing a face, indicating basic differences between skeptics and believers in low-level perceptual decision making processes (e.g. the response criterion or the drift rate; cf. [36]). The more conservative response bias for skeptics compared to believers may indicate that skeptics rely on different internal diagnostic features (e.g. more features or more detailed features) for distinguishing a face from a non-face object compared to believers. An interesting opportunity for future studies would be to more directly investigate the internal representations that skeptics vs. believers use to distinguish different stimulus categories, for instance by using reverse correlation methods [37,38].

In the second experiment it was found that belief in conspiracy theories was associated with differences in visual attention. Typically, in global / local tasks a *global precedence effect* (i.e. faster responses when attending to global compared to local features) and a *global-to-local interference effect* (global stimulus features automatically interfere with attending to stimuli at a local level) can be observed, indicating a dominance of global over local visual processing [39,40]. In the present study, skeptics compared to believers showed a classical global-to-local interference effect, whereas believers compared to skeptics tended to show a stronger local-to-global interference effect. The local-to-global interference effect may indicate that believers had more difficulty in ignoring irrelevant local visual information when making a decision about the global stimulus features. As argued in the introduction, this finding fits with the notion that belief in conspiracy theories is associated with endorsing a ‘tunnel vision’ in which detailed and specific information is selectively processed, while less attention is paid to contextual information. The dominance of local compared to global features could possibly also account for the finding that believers in conspiracy theories hold apparently contradictory views [28], as each specific belief may be considered in isolation without taking into account other beliefs or the more general context. This attentional bias may be related to a process of socialization and education, in which people developed a strategy of selectively focusing or attending to information that confirms their worldview (i.e. confirmation bias, see also: [41,42]).

However, it should be noted that the present study was exploratory (i.e. a priori two different and opposing predictions could be made regarding the relation between conspiracy beliefs and global / local processing). The effects observed in the second study using simple main effects analysis were only marginally significant, which may be related to the relatively small sample size. Therefore, these findings should be interpreted with caution. Future studies are required to confirm the observed relationship to warrant stronger conclusions regarding the cognitive and attentional biases underlying belief in conspiracy theories. Furthermore, in the present study a small relation between conspiracy beliefs and reaction time effects was observed, but the error rates were unaffected. The finding of an effect for reaction time measures but not for the error rates when using the Navon task is a classical finding [31] and previous studies have reported a similar relation between reaction time effects and religious beliefs for instance [1,5]. The reaction time effect reflects the interference of irrelevant visual information on perceptual decision making, which is in turn related to an attentional bias to automatically attend to either global or local visual information [31]. Thus, the reaction time effect does not merely reflect a general congruency effect (i.e. as would be obtained when using a Stroop-task for instance), but is specifically related to the interference of congruent or incongruent visual information at different levels in the visual processing hierarchy.

In the present study, a moderate correlation was observed between paranormal beliefs and belief in conspiracy theories that was comparable in size to previous studies [18,24,25]. It could well be that the observed relation is primarily related to individual differences in education [29,43], ontological confusions [44] and / or an intuitive compared to an analytical thinking style [29,30,45], underlying both the endorsement of conspiracy and paranormal beliefs. In addition, paranormal and conspiracy beliefs could be mutually reinforcing: e.g. the belief that major pharmaceutical companies and governments hide important information about available therapies may motivate someone to seek refuge in alternative medicine. Still, the finding that the local-to-global interference effect was only predicted by conspiracy but not by supernatural beliefs, suggests that these belief types differed to some extent. A key feature of paranormal beliefs it the belief that the mind is somewhat ‘porous’ and that thoughts can travel between persons (i.e. as in mindreading) and between persons and supernatural agents (e.g. as in ‘channeling’; cf. see: van Elk et al., in prep). In contrast, conspiracy beliefs are characterized by attributing intentionality and agency to inanimate objects [26,43] and a tendency to connect apparently unrelated events [28]. By using novel techniques, like network analysis [46], the complex relation between these different constructs and the items that comprise these scales, could be determined in future studies.

Previous studies have shown that both paranormal and conspiracy beliefs are strongly associated with schizotypal personality features [18,47,48,49,50]. It has been found for instance that participants with schizotypal personality features are more prone to detecting meaningful patterns in meaningless noise [51,52] and to making false alarm responses [53,54,55,56]. The present study indicates the importance of the task that is used for making claims about the relation between schizotypy and perceptual biases: whereas people with schizotypal personality features may make more false alarms on a signal detection task, no bias may be observed when using a categorization task as in the present study. Interestingly, schizotypy has also been associated with an exaggerated focus on local compared to global information [57,58]–similar to the effect observed in the present study for belief in conspiracy theories. An important challenge for future studies would be to investigate to what extent the psychological mechanisms that have been described for belief in the paranormal and belief in conspiracy theories are unique or overlap with the neurocognitive mechanisms that are characteristic of schizotypy (e.g. by directly comparing schizoptypical believers and non-believers) and to what extent the perceptual biases observed are task-specific or reflecting a domain-general process (e.g. apophenia; cf. [7]).

In sum, the present study shows that individual differences in prior paranormal and conspiracy beliefs are associated with perceptual decision making and visual attention. These findings extend the growing body of work in this field indicating effects of cultural learning on basic perceptual processes and underlining the importance of individual differences in relation to the study of paranormal and conspiracy beliefs [1,5,9,10,59,60].

## Supporting Information

S1 File This contains the questionnaire and behavioral data that was analyzed in the present study. (XLSX)
